# Preparation and Performance Analysis of Modified Sodium Acetate Trihydrate

**DOI:** 10.3390/ma11061016

**Published:** 2018-06-15

**Authors:** Weisan Hua, Xuelai Zhang, Munyalo Jotham Muthoka, Xingchao Han

**Affiliations:** Institute of Thermal Storage Technology, Merchant Marine College, Shanghai Maritime University, Shanghai 201306, China; weisanhua@yeah.net (W.H.); muthokamunyalo@yahoo.com (M.J.M.); xinchao5456@126.com(X.H.)

**Keywords:** thermal storage materials, sodium acetate trihydrate, pcm, undercooling, phase separation

## Abstract

In order to solve undercooling and phase separation of sodium acetate trihydrate (SAT), experimental screening method was used to select nucleating agents and thickeners that are suitable for SAT, and the optimal ratio was identified. Through screening experiments of nucleating agents, it is found that disodium hydrogen phosphate can be used as an effective nucleating agent for SAT. When the weight content of disodium hydrogen phosphate in SAT is 2%, the degree of undercooling was reduced to approximately 2 K. The addition of 1–1.5% (weight) of xanthan gum (XG) to SAT can effectively inhibit the phase separation. Since the properties of SAT changes after the modification, the corresponding comparison analysis was performed. The results showed that XG has a significant influence on the SAT performance of SAT. With the addition of 1.5 wt % of XG in pure SAT, the latent heat of fusion and solid/liquid volume expansion reduce by 5.2% and 5.4% respectively, and the thermal conductivity and solid/liquid density also decreases accordingly.

## 1. Introduction

Phase change materials (PCMs) can absorb or release a large amount of latent heat when they undergo a phase change. Therefore, they can be widely used in the fields of solar thermal storage, waste heat recovery, building temperature control and electric power peak adjustment [1,2,3,4,5]. Among many medium low temperature PCMs, hydrated salt PCM has the advantages of high heat storage density, good thermal conductivity and low cost. Therefore, these PCMs have been well developed and have become the most promising and important TES materials [6,7,8,9]. SAT is a typical representative of hydrated salt PCM. It has a suitable melting temperature and high latent heat of fusion. The raw material is abundant, and is harmless to the environment. With these advantages, SAT can be widely used in solar heat and waste heat storage [10,11,12,13]. However, similar to most hydrated salts, SAT has serious undercooling and phase separation problems in the melting or solidification process.

Many scholars have conducted relevant mechanisms and experimental studies on the problem of SAT and they have achieved certain results. In 1982, Japanese scientist Takahiro Wada used SAT as a heat storage matrix material to study the nucleation effect of Na_4_P_2_O_7_·10H_2_O addition. Results showed that after adding 0.1 g of Na_4_P_2_O_7_·10H_2_O to 10 g of SAT, SAT does not crystallize at the melting point temperature. However, it crystallizes at 10 °C below the melting point [14]. Later, Takahiro Wada and others studied the effects of preheating on the crystallization of SAT with nucleating agents, as well as the influence on the nucleation process and the failure temperature [15,16,17]. In 2003, Spanish scientist L. F. Cabeza and others discussed the issue of undercooling and phase separation of SAT. It was found that adding 1% nucleating agent Na_2_HPO_4_·7H_2_O in SAT can effectively inhibit undercooling and phase separation phenomena [18]. In 2016, Yuan Yanping reported that Nano-copper (Nano-Cu), which possesses high thermal and electrical conductivity, as an additive, can improve the supercooling properties of sodium acetate trihydrate (CH_3_COONa·3H_2_O, SAT) and enhance its thermal conductivity [19]. In 2017, Mao Jinfeng prepared a new composite phase change thermal storage material by adding a certain amount of nucleating agent, thickening agent, and high thermal conductivity medium. The research results showed that addition of an appropriate proportion of nucleating agent disodium phosphate dodecahydrate and thickening agent carboxymethyl cellulose or gelatin led to a good restrain to the undercooling of SAT, while maintaining the heat storage capacity of the sample, and avoiding the phenomenon of phase stratification at room temperature [20].

From the above literature review, it is found that many scholars have done relevant research on the undercooling and phase separation of SAT. However, there is little related research on the analysis of the influence of nucleating agents and thickeners on the performance of SAT. Parameters like latent heat value of phase change, thermal conductivity, solid-liquid volume change, solid-liquid density and pH are very important for the engineering application of modified SAT. The purpose of this paper is to test the key parameters of modified SAT and analyze the effect of additives on the performance of SAT. The results of the test and analysis can be used for further research and engineering applications.

## 2. Experimental Materials and Methods

### 2.1. Experimental Materials

Sodium acetate trihydrate (SAT, purity ≥ 99.0%) was employed as matrix material in the study. Disodium hydrogen phosphate (DSP), tetrasodium pyrophosphate decahydrate (TPD), anhydrous sodium acetate (SAA), sodium tetraborate decahydrate (STD), sodium metasilicate nonahydrate (SMN) were used as alternative nucleating agents in the experiment. Carboxymethyl cellulose sodium (CMC), Polyacrylic acid sodium salt (PASS), xanthan gum (XG) and palygorskite (PAS) were used as alternative thickeners in the experiment.

### 2.2. Experimental Instruments

The main instruments in the experiment are an electric heating constant temperature water bath (DK-S600, Shanghai Hengping Instrument Factory, Shanghai, China, heating range 0~100 °C, ±0.1 K accuracy), electronic balance (measuring range 0–200 g, ±0.1 mg accuracy), differential scanning calorimetry instrument (DSC, measuring range −170~600 °C, provided by Netzsch Scientific Instruments Trading (Shanghai Co., Ltd, Shanghai, China), Agilent data acquisition instrument (measuring range −200~200 °C, ±0.01 K accuracy), hot disk thermal constant analyzer (TPS500, K-ANALYSIS TRADING (Shanghai Co., Ltd., , Shanghai, China, measuring range 0.005–20 W/mK, ±3% accuracy), thermocouple temperature measurement line (the T-type, measurement range −60–300 °C, ±0.05 K accuracy).

### 2.3. Experiment Methods

#### 2.3.1. Time-Temperature Curve

**Figure 1:**
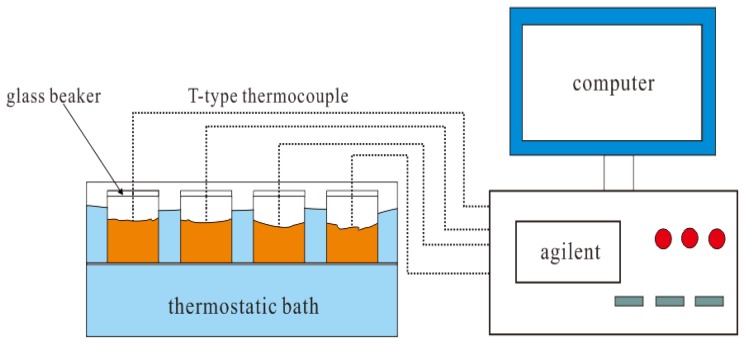
The test system of PCMs’ melting or solidification.

For nucleation experiments, the time-temperature curve method is generally used to judge the nucleation effect. Figure 1 shows a melting and solidification test system for PCM. The system can easily test the time-temperature curve of PCM in melting and solidification. The computer in the system is used to record and save experimental data and Agilent is used for recording real-time data. T-type thermocouple wires are used for temperature signal transmission, and glass beakers are used as PCM containers. The melting heat source of PCM is a constant temperature water bath, and the solidification cooling source is room air.

#### 2.3.2. Analysis of Parameters

6 parts of 30 g of pure SAT samples are set on an electronic balance and then they are put into 6 glass beakers respectively with related marks. Similarly, 0.3 g (1 wt %) of DSP, TPD, SAA, STD and SMN can be calculated by an electronic balance and be placed in a glass beaker with pure SAT samples. The marked T-type thermocouples are inserted into the above-mentioned 6 glass beakers respectively. Then they are placed in a constant-temperature water bath at 70 °C for heating, and the heating lasts for 66 min. After all samples are melted, they are taken out of the thermostatic heating tank and placed in air to cool. In the cooling process of the sample, the ambient temperature is 16 °C. The above experimental operation should be repeated 4 times.

After the addition of nucleating agents and thickeners in pure SAT, many properties will be affected. Therefore, it is necessary to make analysis. Regarding pure SAT and modified SAT, the parameters that need to be compared for analysis include the latent heat of fusion, solid/liquid thermal conductivity, pH, solid/liquid density and solid/liquid volume expansion.

## 3. Results and Discussion

### 3.1. Screening of Nucleating Agents

**Figure 2:**
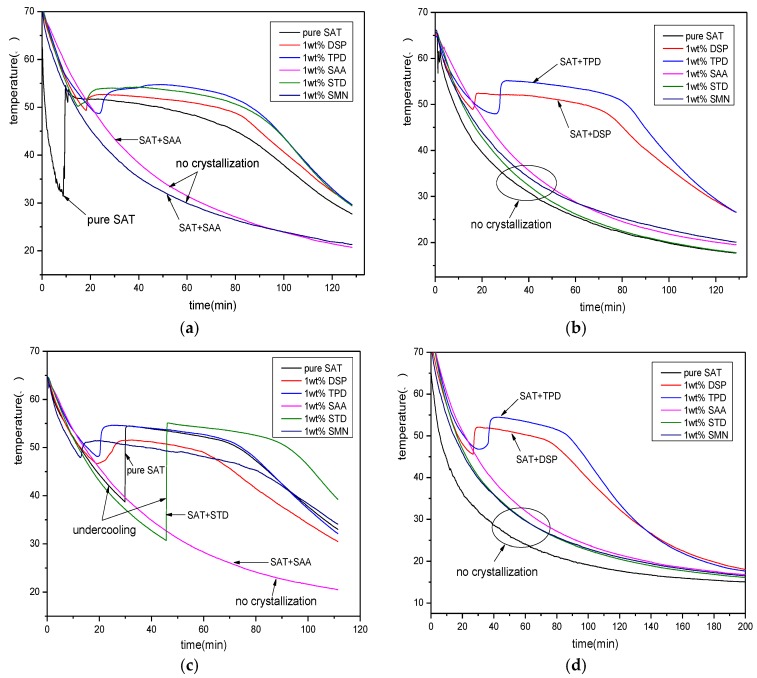
The discharging curves of samples after adding nucleating agents. (**a**) the first discharging curves; (**b**) the second discharging curves; (**c**) the third discharging curves; (**d**) the fourth discharging curves.

From Figure 2, it can be seen that the pure SAT sample appears undercooling during the first discharging process. The temperature of the SAT sample begins to decrease after reaching 32 °C and the degree of undercooling reaches 23 K. During the subsequent three discharging process, the pure SAT sample is always liquid and there is no phase change crystallization. The five samples with nucleating agents appear with different phase change crystallization effects during four discharging processes. Among them, samples with SAA do not undergo phase change crystallization during the four discharging process. It shows that SAA has no nucleating effect on SAT and it is not suitable to be sued as a nucleating agent for SAT. The SAT sample with STD and SMN appears in different degrees of undercooling during the first and third discharging process. There is no phase change crystallization during the second and fourth discharging process. This shows that STD and SMN have a certain nucleation effect on SAT, but the nucleation is very unstable, so they are not suitable as a good nucleating agent for SAT. However, the SAT samples with DSP and TPD have stable crystallization during the four discharging process, and demonstrate good phase-change constant-temperature platform. Therefore, they can be used as an alternative nucleating agent for SAT.

**Figure 3:**
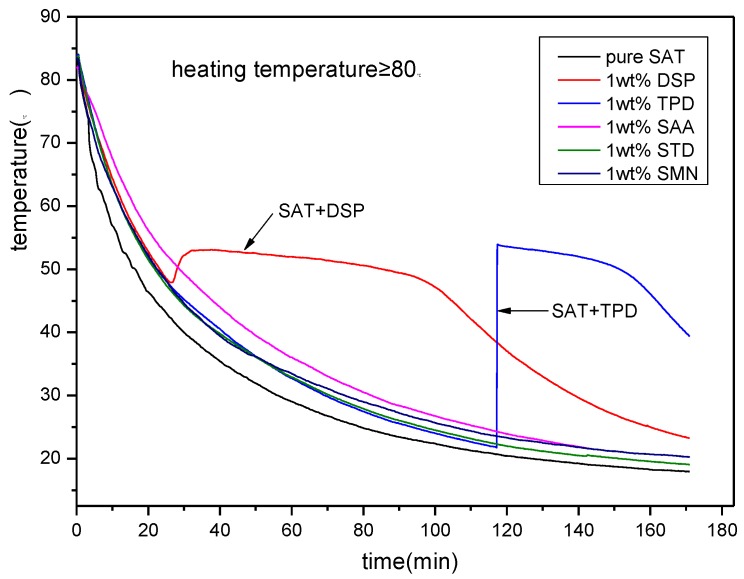
The discharging curves of SAT with nucleating agents at high heating temperature.

The above experimental results show that both DSP and TPD can be used as nucleating agents for SAT. However, the above experiment is performed at a certain temperature (≤75 °C). While the effect of heating temperature on nucleation is not considered. To further screen the best nucleating agent for SAT, a nucleating agent screening experiment at a higher temperature is necessary. Figure 3 shows the discharging process of all SAT samples after heating to 85 °C. From the figure, it can be seem that pure SAT sample and SAT samples with SAA, STD and SMN do not show phase change crystallization during the discharging process. The phase change crystallization started after the temperature of the SAT sample with TPD is reduced to 25 °C and the degree of undercooling reaches 30 K. Regarding the SAT sample with DSP, the phase change crystallization appears steadily, and there is an obvious constant temperature heat release platform. The degree of undercooling is approximately 5 K. This experiment demonstrates that DSP has the best nucleation effect on SAT when the heating temperature is not less than 80 °C.

**Figure 4:**
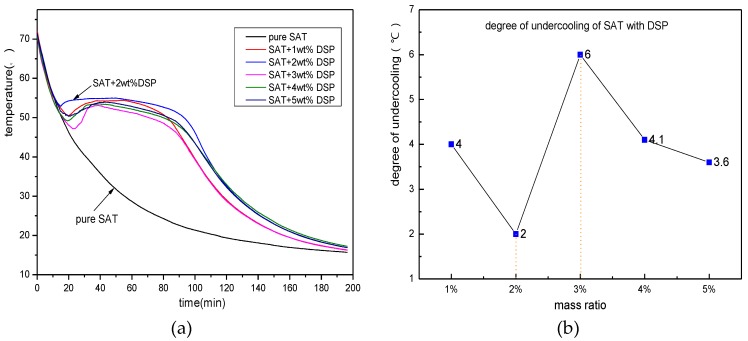
The discharging curves of nucleating agent with different proportions. (**a**) the discharging curves of SAT with DSP; (**b**) distribution of degree of undercooling.

After DSP is selected as an effective nucleating agent for SAT, it is necessary to do different addition ratio screening experiments. Figure 4 shows the discharging process curves of SAT samples with 1, 2, 3, 4 and 5 wt % of the DSP addition ratio. It can be clearly seen from the figure that all SAT samples undergo phase change crystallization, have excellent phase change constant temperature platforms. Among them, when the addition content of DSP is 2 wt %, the nucleation effect is the best. The degree of undercooling is within 2 K, and the phase change constant temperature platform is the longest. When the addition ratio is 1 and 5 wt %, the degree of undercooling of the sample is 4 and 3.6 K respectively. When the addition ratio is 3 and 4 wt %, the degree of undercooling of the sample material is 6 and 4.1 K respectively. The experiment shows that there is no fixed rule between the amount of nucleating agent added and the nucleating effect. The relationship is nonlinear.

### 3.2. The Selection of Thickeners

**Figure 5:**
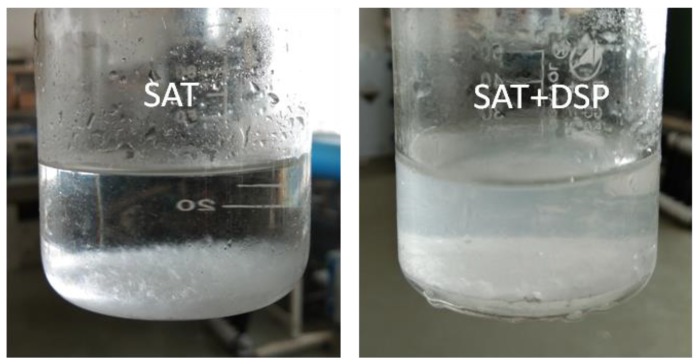
Phase separation and precipitation.

After the pure SAT sample and the SAT sample with nucleating agents undergo five melting/solidification cycles, there were crystallized precipitates and white precipitate at the bottom, as shown in Figure 5. Regarding pure SAT samples, the precipitate is sodium acetate without crystalline water. Regarding SAT samples with DSP, the precipitate is SAT without crystal water and DSP without crystal water.

When phase separation and precipitation in PCM occurs, their thermal storage properties will gradually decline. Therefore, the PCM with a nucleating agent must be thickened to suppress its phase separation and precipitation phenomena. Common thickeners include inorganic thickeners, cellulose ethers, natural polymers, and synthetic polymers. In this experiment, one of the above classes was used as an alternative thickener. They are PAS, CMC, XG and PASS.

**Figure 6:**
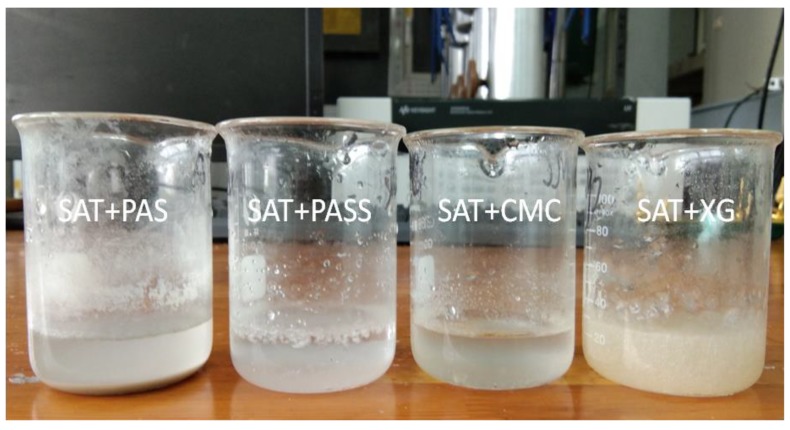
Thickening effect of different thickeners on SAT samples.

According to literature [16], the amount of thickening agent is generally approximately 2 wt %. So this content is used in the experiment to do a preliminary selection of thickeners. Figure 6 shows the thickening effect of SAT samples added with 2 wt % of PAS, PASS, CMC, XG, and it can be seen from the figure that the sample with XG has the best thickening effect when the heating temperature is 75 °C. There is no layer phenomenon, thus it can well inhibit the phase separation of SAT. PASS and CMC have a certain thickening effect. However, after they are placed in a constant temperature for 8 h, the upper part is clear liquid as the majority, while the lower part of the body is turbid liquid. That means PASS and CMC are not suitable as thickeners to SAT. The sample with adding PAS has the worst effect, the viscosity of the liquid SAT sample is not increased. There are precipitates at the bottom of the beaker, so PAS is also not suitable as thickener for SAT.

### 3.3. The Interaction of Nucleating Agents and Thickeners

In the experiments of Section 3.1 and Section 3.2, the effective nucleating agents and thickeners of SAT were selected respectively. However, the interaction effect of the two on SAT needs further study. The experiment of Section 3.1 determines that the effective nucleating agent of SAT is DSP, and its optimal adding proportion is 2 wt %. The experiment in Section 3.2 only determines the effective thickener of SAT, and it does not determine its optimal addition content.

**Figure 7:**
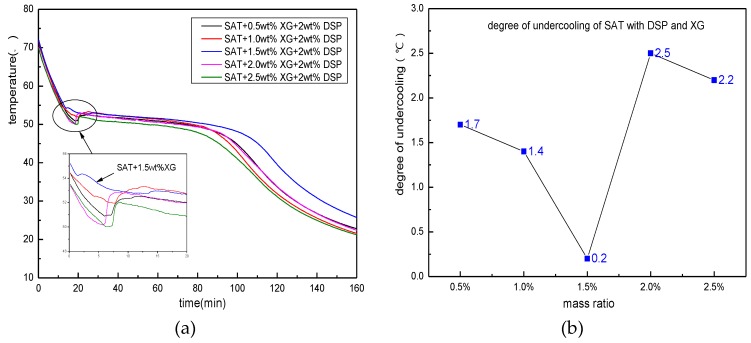
The nucleation effect of thickeners on matrix material. (**a**) the discharging curves of SAT with XG and DSP; (**b**) distribution of degree of undercooling.

Figure 7 shows the heating/cooling curve of five parts of SAT samples which contain 2 wt % of the nucleating agent and 0.5, 1, 1.5, 2, 2.5 wt % of thickeners. It can be seen from the cooling process of the sample in the figure that the degree of undercooling of the sample shows a downward tendency when the amount of XG is increased from 0.5 to 1.5 wt %. When the content of XG is 1.5 wt %, the degree of undercooling decreases to 0.2 K. However, when the amount of XG increases from 1.5 to 2.5 wt %, the degree of undercooling of the sample increases to 2 K or above. When the amount of addition is 2 wt %, the degree of undercooling is 2.5 K, which is greater than the degree of undercooling without the addition of XG. It can be seen that a certain proportion of thickeners can help the nucleating agent to hinder the SAT’s undercooling. The combined effects of the two can minimize the degree of undercooling. However, the amount of thickener should not be too large. The reason for this phenomenon can be explained by the mechanism of the thickener as below. The thickener has a strong water absorption effect. After it is mixed with the SAT, it will absorb part of the crystal water of the SAT, making itself very viscous. After the XG absorbs water, the mucus will cover the nucleus of the SAT, inhibiting its crystallization and growth, and cause the SAT’s degree of undercooling to increase. In addition, if the amount of thickener is too large, it will affect the heat storage density and heat transfer of the SAT. Therefore, the addition amount of the thickener should not be too large, and should be controlled at approximately 1 to 1.5 wt %.

### 3.4. Analysis of DSC

**Figure 8:**
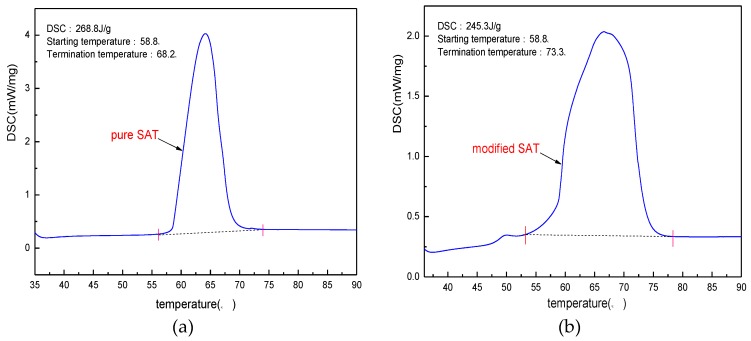
DSC curves of pure SAT and modified SAT. (**a**) pure SAT; (**b**) modified SAT.

According to the experimental results from Section 3.1 to Section 3.3, the optimal ratio of the modified SAT is determined as 96.5 wt % of SAT, 2 wt % of DSP and 1.5 wt % of XG. The modified SAT sample is prepared according to the ratio. The phase transition characteristics of the pure SAT and the modified SAT are analyzed by a differential scanning calorimetry instrument. The results of the test are shown in Figure 8.

From the perspective of mass fraction, the maximum loss of latent heat value is 3.5% after adding thickeners and nucleating agents to the SAT sample. However, the DSC test results in Figure 8 show that after DSP and XG are added to the SAT sample, the latent heat decreases from 268.8 to 245.3 J/g, with a decrease of 8.7%. This value is different from the theoretical analysis value and the difference is 5.2%. After analysis and experimental observation, it is found that the main cause of the difference is the water absorption of XG. XG has a strong hydrophilicity. When it is mixed with liquid SAT, it will absorb the crystal water of SAT, resulting that part of SAT loses crystal water. The SAT without crystal water cannot be reduced and eventually become anhydrous sodium acetate. The anhydrous sodium acetate has no phase change within the test temperature range, which results in a drop in the DSC value of the sample. This conclusion can also be verified from the DSC curve of the modified SAT. The initial temperature of the phase transition of the modified SAT sample is the same as that of the pure SAT: both are 58.5 °C. However, the phase transition termination temperature is 5.1 K higher than that of the pure SAT sample. The phenomenon is due to the performance of the SAT after losing crystal water.

### 3.5. Analysis of Thermal Conductivity

**Figure 9:**
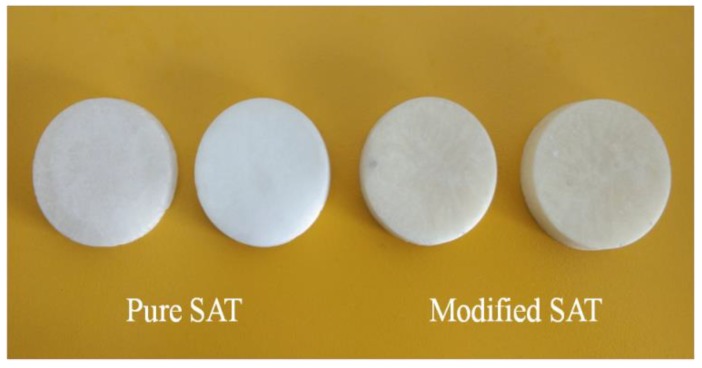
Solid SAT samples.

Thermal conductivity is an important index to present heat transfer performance of materials. Additive types and components have a significant influence on the thermal conductivity of the base material. Therefore, it is necessary to do a comparison test of thermal conductivity of pure SAT and modified SAT. According to the test principle of the hot disk thermal constant analyzer, when the thermal conductivity of the solid PCM is tested, it is necessary to compress the sample into a round cake-shaped module as shown in Figure 9. Before testing the thermal conductivity of the sample, the surface of the sample is guaranteed to be smooth. When testing the thermal conductivity of liquid PCM, the sample must be completely melted. To ensure the accuracy of the test, the interval between two adjacent tests is at least 30 min.

Table 1 shows the solid/liquid thermal conductivity values of pure SAT and modified SAT. Comparing the data in the table, it can be noted that the solid/liquid thermal conductivity of the modified SAT is lower than that of the pure SAT. In the experimental process of Section 3.3, it has been found that the SAT sample has volume expansion and bubble inclusion after XG addition. This phenomenon is directly related to the decrease of the solid/liquid thermal conductivity of the modified SAT. When XG absorbs water, the viscosity of the SAT sample will increase. Thus it degrades the fluidity of the liquid SAT, resulting in a decrease in the convection heat transfer capacity. At the same time, the high viscosity of XG makes it difficult for the bubbles, resulting from the stirring or heating in the SAT sample, to escape. It results in many micro pores contained in the SAT sample, which lead to a decrease in the thermal conductivity of the solid SAT sample.

### 3.6. Analysis of pH Tests

**Figure 10:**
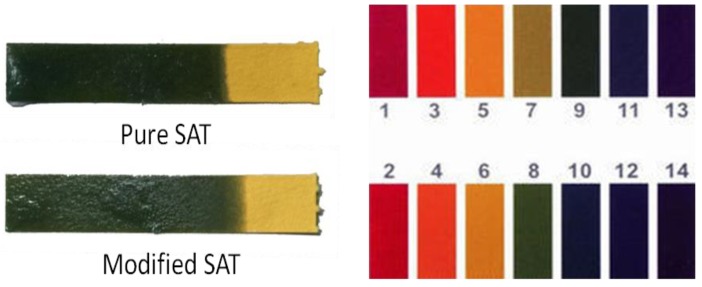
pH values of the pure SAT and modified SAT.

The pH of PCM is directly related with corrosiveness. If the prepared PCM is a strong acid or a strong base, the material has a large corrosivity to the packaging container. Therefore, when selecting and preparing a composite PCM, its pH must be measured. In this experiment, the pH test paper is used to test the pH of pure SAT and modified SAT. The test results are shown in Figure 10. Comparing the test results with the standard pH chart, it can be found that both the pure SAT and the modified SAT are medium-weak bases with a pH of 8. According to the literature [21] and the test results of pH, stainless steel is recommended as the packaging material of modified SAT.

### 3.7. Analysis of the Volume Change Rate and Solid-Liquid Density

There are changes in density and volume during melting and solidification of both pure SAT and modified SAT. So it is necessary to do accurate measurements during preparation. In this experiment, a very simple measurement method is used. Only a constant temperature water bath, electronic balances, and graduated cylinders are used to measure the material density and volume change rate.

The measurement procedures are as follows: (1) 30 g pure SAT samples and modified SAT samples are respectively weighed by an electronic balance. Then they are put into the marked cylinder; (2) After the sample material is stirred in the graduated cylinder uniformly, it is placed in a constant temperature water bath at 70 °C; (3) After the sample material in both cylinders is completely melted, the volume V_l_ in the two cylinders at this time is recorded; (4) After the volumetric reading of the liquid sample material is completed, two measuring cylinders are taken out of the constant temperature bath and be placed at room to cool naturally; (5) After the pure SAT sample and the modified SAT sample in the measuring cylinder are completely solidified, the upper surface of the sample is broken with a small metal rod, so that the space on the upper part of the sample can be exposed due to solidification shrinkage. Then, 20 mL of silicone oil (V_o_ = 20 mL) is poured, and the volume V_a_ in the measuring cylinder is recorded. In order to ensure the accuracy of the test results, each parameter needs to be measured more than three times, with the average value is taken as the test result; (6) The V_l_, V_a_, V_o_ and m_s_ are calculated by Equations (1)–(4) to obtain the volume expansion rate and solid/liquid density of pure SAT and modified SAT.

(1) $$\rho_{l}=\frac{m_{s}}{V_{l}}$$

(2) $$\rho_{s}=\frac{m_{s}}{V_{a}-V_{o}}$$

(3) $$V_{s}=V_{a}-V_{o}$$

(4) $$\Delta V=\frac{V_{l}-V_{s}}{V_{s}}$$

Among them,

$\rho_{l}$—liquid density;
m_s_—sample mass;
V_l_—liquid volume;
$\rho_{s}$—solid density;
V_s_—solid volume;
V_o_—the volume of silicone oil in the measuring cylinder;
ΔV—volume change rate of solid and liquid;
V_a_—The sum of the volume of solid phase change material and silicone oil.

The test process ignores the volatility loss of the phase change materials and the silicone oil. The solid/liquid density of the phase change material can be calculated by Equations (1) and (2). The volume expansion rate can be calculated by Equations (3) and (4). All measurements and results are summarized in Table 2:

According to the analysis in Section 3.4, the use of thickeners will increase the viscosity and volume expansion of the modified SAT. The increase of the viscosity will reduce the free space of the matrix material, reducing the solid-liquid volume change rate of the modified SAT. The volume expansion reduces the fixed-mass modified SAT density. Based on the data in Table 2, it can be seen that the average solid-liquid volume change rate of pure SAT is 16.3%; The average volume change rate of modified SAT is 10.9%, and the solid-liquid volume change rate of SAT after modification is reduced by 5.4%. Regarding the solid-liquid density, the value of the modified SAT sample is less than that of pure SAT. The decrease of the solid-liquid density means that the energy density of the material also decreases: it is undesirable to the practical applications of the material.

## 4. Conclusions

To solve the problem of undercooling and phase separation of pure SAT, experimental screening method was utilized to screen its effective nucleating agents and thickeners. At the same time, the properties of the modified SAT are analyzed and the following conclusions are obtained:

(1) DSP is an effective nucleating agent to SAT, which can effectively suppress the degree of undercooling of SAT. When the DSP’s addition content is 2 wt %, the degree of undercooling of SAT can be reduced to 2 K.
(2) Based on thickening agent screening experiments, it is found that XG can be used as an effective thickener for SAT. It can effectively solve the problem of phase separation of pure SAT. When the mass content of XG is between 1 and 1.5 wt %, thickening and dispersing effects are optimal and there is no stratification. With the combined effect of thickener and nucleating agents, the degree of undercooling of modified SAT can be reduced to within 1.5 K. The recommended mass composition of modified SAT is 96.5 wt % SAT, 2 wt % DSP, 1.5 wt % XG.
(3) Through the comparative tests of parameters, it is found that the thickener is the main factor that affects the performance of modified SAT. After thickeners are added to pure SAT, its latent heat, solid/liquid volume expansion rate, solid/liquid density and thermal conductivity all decrease accordingly. Therefore, in actual applications, the mass content of XG is between 1 and 1.5 wt %.

## Figures and Tables

**Table 1:** Thermal conductivity values of solid liquid materials.

Material Name	Solid Thermal Conductivity (W/m·K)	Average Value (W/m·K)	Liquid Thermal Conductivity (W/m·K)	Average Value (W/m·K)
SAT	0.8945	0.9015	1.51	1.51
	0.9130		1.53	
	0.8971		1.49	
SAT + DSP + XG	0.6895	0.6818	1.21	1.21
	0.6885		1.18	
	0.6675		1.25	

**Table 2:** Volume expansion rates and densities for pure SAT and modified SAT.

Material Name	V_l_ (mL)	V_a_ (mL)	V_o_ (mL)	m_s_ (g)	ΔV	$\rho_{s}/\rho_{l}\left(kg/m^{3}\right)$
	21.0	38.0	20	30	16.7%	
SAT	21.5	38.5	20	30	16.2%	1.636/1.406
	21.5	38.5	20	30	16.2%	
	23.5	41.5	20	30	9.3%	
SAT/DSP/XG	24.0	41.5	20	30	11.6%	1.406/1.268
	23.5	41.0	20	30	11.9%	
